# Evaluation of the 2021–2024 Implementation of the Fresh Start Enhanced Food Is Medicine Intervention Among Rural, Underinsured People With Type 2 Diabetes

**DOI:** 10.5888/pcd23.250471

**Published:** 2026-07-23

**Authors:** Lauren R. Sastre, Brandon Stroud, Raven Breinholt, Alessio Fratacangeli, Sarah Elliott, Amy Turner, Bhibha M. Das, Sissy Lee-Elmore, Yolanda Cristiani, Jennifer A. Buxton, Belen Rogers

**Affiliations:** 1 Department of Nutrition Science, College of Allied Health Sciences, East Carolina University, Greenville, North Carolina; 2 Department of Health Education and Promotion, College of Health and Human Performance, East Carolina University, Greenville, North Carolina; 3 Department of Kinesiology, College of Health and Human Performance, East Carolina University, Greenville, North Carolina; 4 WATCH Clinic, Goldsboro, North Carolina; 5 Hope Clinic, Bayboro, North Carolina; 6 Cape Fear Clinic, Wilmington, North Carolina; 7 Food Bank of Central & Eastern North Carolina, Raleigh, North Carolina

## Abstract

**Purpose and Objectives:**

We aimed to evaluate the feasibility, acceptability, and impact of the Fresh Start produce prescription (FSPRx) intervention among rural, underinsured people with type 2 diabetes.

**Intervention Approach:**

The 20-week FSPRx intervention was implemented in 15 counties in eastern North Carolina to provide 5 to 7 pounds of produce at 9 group classes and individual, telephone-based health coaching.

**Evaluation Methods:**

We evaluated feasibility by reviewing program records, acceptability by reviewing surveys and interviews, and impact by reviewing validated pre–post questionnaires on knowledge, skills, and behavior and retrospective medical record review (glycated hemoglobin A_1c_ [HbA_1c_]). Quantitative data were analyzed by using descriptive statistics, the Wilcoxon signed-rank test, paired sample *t* tests, repeated-measures analysis of variance (ANOVA), and multiple linear regression. We analyzed interview transcripts by inductive content analysis for themes.

**Results:**

Approximately half of enrolled participants (N = 414) completed pre–post measures (n = 225) and actively participated (n = 170). Participants were majority female (65.4%) and were White (36.5%) or Black (34.3%). Program satisfaction was 96.5%. Participation was moderate for coaching encounters and class attendance (mean [SD], 3.1 [4.5] encounters; 2.0 [2.9] classes, respectively). Food literacy and consumption of fruits, vegetables, and whole grains increased significantly. Glycemic control (mean HbA_1c_) improved significantly, with the greatest decreases among participants with 4 or more classes and 4 or more health coaching encounters (0.44% in the full analytic sample). Group classes were the strongest predictor of improved HbA_1c_ (B = −0.07, *P* = .06). Interest was high for flexible remote education (program notebook, 97.1%).

**Implications for Public Health:**

Strengths of the FSPRx intervention were acceptability and impact, while feasibility was moderate. Although participation in group classes was the strongest predictor of improved HbA_1c_, attendance was limited. Research is warranted to identify optimal integration of educational and behavioral support in Food Is Medicine programs, especially for rural populations.

**Summary**

**What is already known on this topic?**

Food Is Medicine interventions offer potential to improve food security, nutrition, and health outcomes.

**What is added by this report?**

Best practices for behavior and education support have not yet been identified, and Food Is Medicine interventions have also been lacking in rural areas.

**What are the implications for public health practice?**

Integration of theory-grounded, food literacy–centered education as part of Food Is Medicine interventions offers potential to optimize impact on nutrition and health outcomes.

## Introduction

Approximately 14.7% of US adults live with diabetes (1). Diabetes is associated with an increased risk of blindness, kidney disease, hospitalization, and annual health care costs up to $12,022 per person (1). Successful diabetes self-management necessitates addressing a range of individual behaviors that depend on resources internal and external to the health care system. While preventing diabetes is a critical role of public health, tertiary preventive programming is also warranted to reduce comorbidities and complications.

Diabetes self-management education (DSME) improves glycemic control, emphasizing regular glucose monitoring, healthy lifestyle, mental health, medication adherence, screenings, and medical care (2). The benefits of maintaining a healthy lifestyle, especially a nutrient-dense, carbohydrate-controlled dietary pattern can improve glycemic control and reduce complications and comorbidities (3–11). Despite the benefits of DSME and nutrition on glycemic control, not all people have adequate access to DSME or healthy food.

People residing in rural areas, particularly the rural southeastern US, are more likely than others to have diabetes while also having limited access to health care and health-promoting resources such as nutritious food (12–17). Food insecurity, defined as limited access to healthy food, is a concern for people with diabetes because it is associated with low diet quality, high carbohydrate consumption, and reduced capacity to maintain glycemic control (18–22). To address poor diet quality and related health outcomes, Food Is Medicine (FIM) initiatives, such as produce prescription (PRx) programs, connect people with healthy food (eg, fresh fruits and vegetables) and have demonstrated potential to improve glycemic control (23–26). While Food Is Medicine programs are promising, few have been implemented in rural areas or the rural southeastern US, and they have not included diabetes or food-centered education (23–26). Integration of food literacy education may be warranted because food literacy (eg, nutrition knowledge, use of food labels, budgeting, meal planning, culinary skills) predicts diet quality (27,28).

Comprehensive Food Is Medicine interventions that moderate the complex barriers to diet quality and glycemic control (eg, access to DSME, food literacy education) should be implemented to enhance public health efforts to improve health outcomes among people with diabetes. The Fresh Start produce prescription (FSPRx) program was developed to address gaps in previous Food Is Medicine PRx interventions while addressing the needs of rural eastern North Carolina communities, which are characterized by a high prevalence of type 2 diabetes, food insecurity, and limited socioeconomic and health care resources (26,29–33).

## Purpose and Objectives

The purpose of this study was to evaluate the feasibility, acceptability, and impact of the FSPRx intervention among rural, underinsured people with type 2 diabetes in 15 rural counties in eastern North Carolina. The FSPRx built on evaluation findings of a pilot PRx intervention, which identified the need and desire for more direct, intensive education and behavior support, thus leading to the integration of group classes and 1-on-1 telephone-based health coaching (34,35). The objectives of this study were to

1. Evaluate feasibility through the evaluation of group class attendance logs, health coaches’ records of encounters, and produce distribution logs
2. Evaluate acceptability through group class surveys, end-of-program surveys, and interviews exploring experience, perceived benefits, and satisfaction; and identify preferences and needs for future iterations
3. Evaluate the impact on food literacy and diet quality and clinical outcomes (glycated hemoglobin A_1c_ [HbA_1c_]) through validated preintervention and postintervention surveys and a retrospective medical record review.

## Intervention Approach

FSPRx was designed to support communities in eastern North Carolina and address problems (food insecurity, poverty, diabetes, lack of access to health care) identified in previous studies (26,29–33).

### Building on a previous PRx intervention

**Figure 1:**
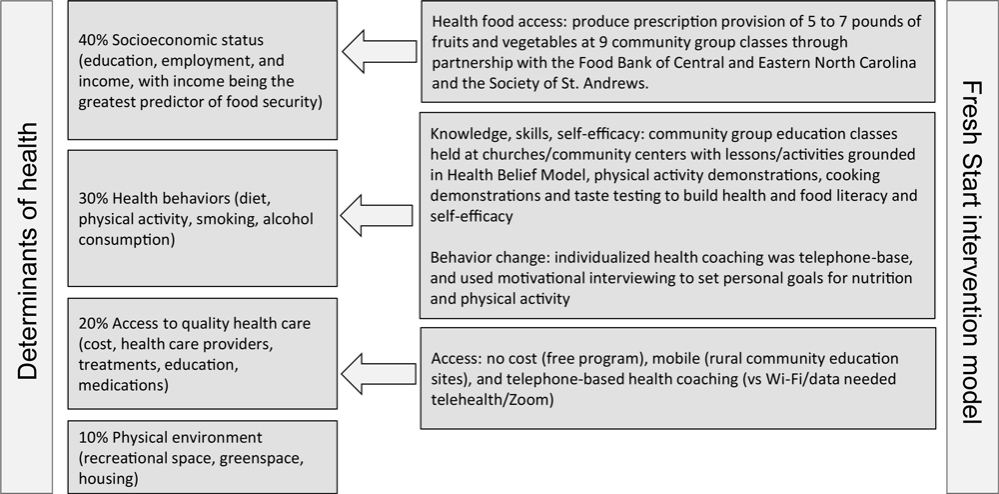
Theoretical model for the Fresh Start produce prescription intervention, or FSPRx, rural eastern North Carolina, 2021–2024. The determinants of health were based on Hood et al (39).

FSPRx built on our experience from the 24-week, delivery-based program, Healthy Eating and Active Lifestyle to Enhance Diabetes management (HEALED) (34,35). HEALED demonstrated significant improvement in HbA_1c_ levels among participants; however, low levels of engagement in indirect, passive nutrition and health education (eg, handouts) resulted in no effect on food literacy (34,35). Participants reported a desire for intensive, direct health and nutrition education with an emphasis on culinary support (34,35). FSPRx educational content, messaging, and approaches were guided by the Health Belief Model (37), which has been shown to improve dietary intake and diabetes self-management (38). Education was delivered through in-person group classes followed by 1-on-1 telephone-based health coaching support. The full, evidence-based theoretical model of the FSPRx intervention targeted established mediators on health outcomes (39) through evidence-based approaches tailored to meet community needs (Figure 1).

**Table:** 

Determinants of health	Fresh Start interventional model
40% Socioeconomic status (education, employment, and income, with income being the greatest predictor of food security	Health food access: produce prescription provision of 5 to 7 pounds of fruits and vegetables at 9 community group classes through partnership with the Food Bank of Central and Eastern North Carolina and the Society of St Andrews.
30% Health behaviors (diet, physical activity, smoking, alcohol consumption)	• Knowledge, skills, self-efficacy: community group education classes held at churches/community centers with lessons/activities grounded in Health Belief Model, physical activity demonstrations, cooking demonstrations and taste testing to build health and food literacy and self-efficacy • Behavior change: individualized health coaching was telephone-base, and used motivational interviewing to set personal goals for nutrition and physical activity
20% Access to quality health care (cost, health care providers, treatments, education, medications)	Access: no cost (free program), mobile (rural community education sites), and telephone-based health coaching (vs Wi-Fi/data needed telehealth/Zoom)
10% Physical environment (recreational space, greenspace, housing)	—

### Eligibility, clinical partners, recruitment, and enrollment

Eligibility criteria for FSPRx included 1) being an adult aged 18 to 64 years without health insurance, 2) a medical diagnosis of prediabetes or type 2 diabetes, and 3) ability to provide informed consent in English or Spanish. Clinical partnerships through the North Carolina Association of Free and Charitable Clinics Access East were essential to recruitment and evaluation. Implementation was staggered with 2 clinics in year 1 (2021–2022), 4 clinics in year 2 (2022–2023), and 5 clinics in year 3 (2023–2024). Clinical partners supported patient referrals through 1) providing lists of all active patients with diabetes, 2) reviewing electronic health records to flag patients who could be referred to the FSPRx, 3) following up to connect these patient referrals to the research team, and 4) recruiting patients in face-to-face encounters in waiting rooms. Patients were scheduled for in-person enrollment appointments on site at the locations of clinical partners. During enrollment, patients provided written informed consent, were assigned an anonymous identification code (ID), completed a preintervention survey, and were encouraged to attend at least 4 group classes and participate in at least 4 health coaching encounters.

### Community-based group education classes and partners

During the 20-week intervention, 9 in-person group classes were provided every other week in the evening at local community sites (eg, rural churches, a local community center). Community partners provided education. Group class included 1) hands-on DSME and food literacy education, 2) physical activity demonstrations, and 3) cooking demonstrations with taste testing. Details on curriculum are available elsewhere (40).

### Health coaching — recruitment, training, and support

One-on-one behavioral support and goal setting were provided by health coaches, who were volunteer undergraduate students trained and supervised by faculty researchers with expertise in nutrition and exercise science. Training for health coaches included knowing and understanding nutrition and physical activity guidelines, motivational interviewing, and SMART (specific, measurable, achievable, relevant, timebound) goal setting. Goals focused on increasing intake of fruits, vegetables, and whole grains, with a focus on nonstarchy vegetables; decreasing intake of fried, high-sugar, and high-sodium foods; and increasing physical activity. Frequency of health coaching encounters was determined by participants, with a target of at least 1 weekly telephone check-in. Details of encounters (date, summary, and notes on goal setting) were recorded in a Research Electronic Data Capture (REDCap) tool built for FSPRx.

### PRx and food partners

Participants were provided a mixed selection (15–25 lb) of fresh produce at each of 9 group classes. This design was built on findings from HEALED, which demonstrated that exposure to a range of produce along with nutrition support (eg, education on health benefits) increases acceptance, willingness to try new foods, and dietary diversity (34,35). Produce was provided at no cost in collaboration with the Food Bank of Central and Eastern North Carolina’s Greenville branch, which provided more than 10,000 pounds of produce during the program, with supplemental produce directly from farms in partnership with the Society of St. Andrew.

## Evaluation Methods

**Figure 2:**
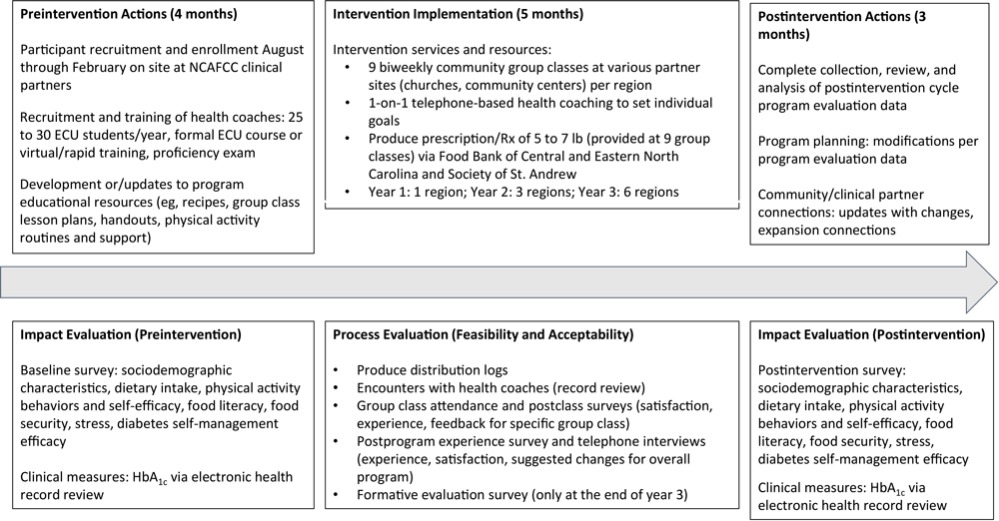
Annual activities for the Fresh Start produce prescription intervention, or FSPRx, rural eastern North Carolina, 2021–2024. Abbreviations: ECU, Eastern Carolina University; NCAFCC, North Carolina Association of Free and Charitable Clinics.

We conducted a mixed-methods implementation evaluation. The intervention and all materials were approved after review by the University and Medical Center Institutional Review Board at East Carolina University (UMCIRB: #21–001619). All data were collected and managed in REDCap, a HIPAA–secure, web-based application designed to support data capture for research studies. Program participation and outcomes were tracked and recorded by the participants’ IDs assigned at enrollment. We summarized our evaluation measures and timeline (Figure 2).

**Table:** 

Timeline		
**Preintervention actions (4 months)** • Participant recruitment and enrollment August–February on site at NCAFCC clinical partners • Recruitment and training of health coaches: 25–30 ECU students/year, formal ECU course or virtual/rapid training, proficiency exam • Development or/updates to program educational resources (eg, recipes, group class lesson plans, handouts, physical activity routines and support)	**Intervention implementation (5 months)** Intervention services and resources: • 9 biweekly community group classes at various partner sites (churches, community centers) per region • 1-on-1 telephone-based health coaching to set individual goals • Produce prescription/Rx of 5–7 lb (provided at 9 group classes) via Food Bank of Central and Eastern North Carolina and Society of St. Andrew • Year 1: 1 region; Year 2: 3 regions; Year 3: 6 regions	**Postintervention actions (3 months)** • Complete collection, review, and analysis of postintervention cycle program evaluation data • Program planning: modifications per program evaluation data • Community/clinical partner connections: updates with changes, expansion connections
**Impact evaluation (preintervention)** • Baseline survey: sociodemographic characteristics, dietary intake, physical activity behaviors and self-efficacy, food literacy, food security, stress, diabetes self-management efficacy • Clinical measures: HbA_1c_ via electronic health record review	**Process evaluation (feasibility and acceptability) ** • Produce distribution logs • Encounters with health coaches (record review) • Group class attendance and postclass surveys (satisfaction, experience, feedback for specific group class) • Postprogram experience survey and telephone interviews (experience, satisfaction, suggested changes for overall program) • Formative evaluation survey (only at the end of year 3)	**Impact evaluation (postintervention)** • Postintervention survey: sociodemographic characteristics, dietary intake, physical activity behaviors and self-efficacy, food literacy, food security, stress, diabetes self-management efficacy • Clinical measures: HbA_1c_ via electronic health record review

### Objective 1: Evaluation of feasibility

We evaluated feasibility by assessing attendance at group classes, provision of produce, and encounters with student health coaches. All tracking used participant IDs.

### Objective 2: Evaluation of acceptability

To examine acceptability, we administered surveys at the end of each group class and a final postintervention survey via telephone. Most questions used a 5-point Likert scale (eg, strongly agree, agree, neutral, disagree, strongly disagree). Group class surveys focused on satisfaction, knowledge gained, and changes in behavior, and open-ended questions explored favorite parts of classes, areas for improvement, and suggestions. The final postintervention survey, administered at the end of each intervention cycle, included closed-ended questions on overall program satisfaction, satisfaction with each of the 3 intervention components, use of recipes and produce, motivation for enrolling, and perceived health and nutrition impact. Open-ended questions explored changes in vegetable preferences, barriers to using produce, favorite aspects, and areas for improvement.

In years 1 (2022) and 2 (2023), we conducted semistructured interviews. Patients were invited to be interviewed when the postintervention surveys were collected. All interviews were recorded via Rev (Rev.com, Inc) and transcribed verbatim. Interviews ranged from 10 to 60 minutes; most lasted 30 minutes. At the end of year 3, in a formative evaluation, we conducted a telephone survey among participants to examine interest in new models of implementation, education approach, and resources (eg, program notebook, social media, website) and potential additional resources or referrals (eg, mental health, dental, primary care, physical therapy).

### Objective 3: Evaluation of impact on nutrition and health outcomes

The preintervention survey (in person at enrollment) and the final postintervention survey (via telephone) were identical and included the following components: 1) questions on sociodemographic characteristics, 2) the validated Hunger Vital Sign 2-question screener for food insecurity risk (41), 3) the validated Rapid Eating and Activity Assessment for Participants food frequency questionnaire (42) to examine dietary patterns, and 4) a validated 19-item tool to assess food literacy (43). This tool scored answers to questions of perceived self-efficacy on a scale of 1 to 7; scores were summed for food literacy subconstruct scores (eg, budgeting, shopping, meal planning, resourcefulness) and for a total food literacy score (ranging from 19 to 133) (36,43). HbA_1c_ data were collected by clinical staff during routine clinical visits, extracted through retrospective record review, and reported to the nearest 0.1%, with pre–post measures collected within 3 months before and after the intervention.

### Analysis

#### Quantitative data analysis

We used descriptive statistics (frequencies, percentages, and mean [SD]) to describe sociodemographic characteristics, group class attendance, health coaching encounters, and closed-ended survey responses. We performed bivariate analyses, tabulating program participators (patients with full preintervention and postintervention data) versus nonparticipators (patients with preintervention data, no group class attendance, and ≤1 health coaching encounter) across the following characteristics: sex, age, race, ethnicity, use of SNAP (Supplemental Nutrition Assistance Program), employment status, food pantry use, food security status, preintervention total food literacy scores, and HbA_1c_ We used Pearson χ^2^ tests, *t* tests, and Mann–Whitney *U* tests to examine potential differences (eg, baseline data, diet quality) between participators and nonparticipators that might bias study outcomes.

To measure impact, we used Wilcoxon signed-rank tests to examine changes in diet quality (n = 143). We used paired sample *t* tests to examine changes from preintervention to postintervention in total food literacy scores (n = 142) and HbA_1c_ (n = 225). We used McNemar tests to examine the proportion of people who moved from uncontrolled HbA_1c_ (≥7.1) to controlled HbA_1c_ (<7.0) from preintervention to postintervention. A multiple linear regression model was then used to examine predictors of postintervention HbA_1c_ while controlling for baseline HbA_1c _and demographic covariates (age, sex, race, and ethnicity). Independent variables included health coaching and group class participation (coded as continuous variables reflecting number of completed sessions). To evaluate whether improvements differed by program engagement level, we conducted a repeated-measures analysis of variance (ANOVA) with time (pre vs post) as the within-subjects factor and program engagement group (health coaching and group class participation targets) as the between-subjects factor. Participants were categorized as low engagement (≤4 group class attended and ≤4 health coaching encounters), health coaching–only target met (≥4 health coaching encounters but ≤4 group class attended), group class–only target met (≥4 group class attended), or both targets met (≥4 health coaching encounters and ≥4 group classes). The threshold 4 was selected because it was an identified threshold to achieve behavior change in a previous study (44). Participants were aware of this target and encouraged both during enrollment and throughout the program to attend 4 group classes and have at least 4 encounters with their health coach.

Statistical significance was set at *P* < .05. All quantitative data were analyzed using SPSS version 31.0 (IBM Corporation). A total of 414 participants enrolled in the program. Sociodemographic data were available for 315 participants (76.1%). HbA_1c_ values were available preintervention and postintervention for 225 participants. We conducted HbA_1c_ analyses for both the full analytic sample (n = 225) and a meaningful program engagement subsample (n = 170) to capture both overall program effectiveness and outcomes among participants with meaningful engagement. The full analytic sample included all participants with available preintervention and postintervention data, regardless of participation level, including people who did not attend any group classes or participated minimally (eg, 1 health coaching encounter). We restricted the meaningful program engagement analysis to participants who demonstrated true program engagement, defined as attending 2 or more health coaching encounters or 1 or more group classes. Participants with missing sociodemographic data were retained in analyses if participation or outcome data were available. The full analytic (n = 225) and meaningful engagement (n = 170) paired samples were sufficient to support the planned inferential analyses and detect meaningful changes in HbA_1c_; therefore, the sample sizes were adequately powered to produce meaningful results. Paired sample *t* tests and McNemar tests were conducted on both the full analytic and meaningful engagement sample. While multiple linear regression analyses were conducted on only the full analytic sample, we conducted repeated-measures ANOVA on the meaningful-engagement sample to examine the effect of participation on HbA_1c_.

### Qualitative data analysis

We used inductive content analysis to review semistructured interviews to identify key themes and subthemes related to participant experiences and program perceptions. Members of the research team (L.R.S., B.S., S.E., A.T.) independently reviewed and coded 3 data-rich transcripts and developed independent preliminary codebooks. The team met to discuss the codebooks and created a single consensus codebook. Then, each research team member individually coded each transcript and met to discuss any discrepancies to reach a consensus on the final codes for each transcript.

## Results

A total of 414 participants enrolled in FSPRx from 2021–2024. Of these, 315 (76.1%) provided baseline sociodemographic data. Of the 315 participants with baseline sociodemographic data, 65.4% were female, 36.5% White, 34.3% Black, 53.3% unemployed, and 44.4% food insecure (Table 1). Mean (SD) age was 52.3 (9.1) years. People with uncontrolled HbA_1c_ (vs uncontrolled) at baseline were significantly more likely to participate in the program (χ^2^_1_ = 4.5, *P* = .03). Females (vs males) were also more likely to participate (χ^2^_1_ = 4.33, *P* = .03). We observed no other differences in participation by sociodemographic characteristics.

**Table 1:** Sociodemographic Characteristics of Rural Medically Underinsured Participants With Type 2 Diabetes (n = 315) in the Fresh Start Produce Prescription Intervention, Eastern North Carolina, 2021–2024

Characteristic	No. (%)^a^
**Sex**	
Female	206 (65.4)
Male	109 (34.6)
**Age, mean (SD), y**	52.3 (9.1)
**Race**	
Asian	3 (1.0)
American Indian or Alaska Native	2 (0.6)
Black or African American	108 (34.3)
Native Hawaiian and Other Pacific Islander	0
White	115 (36.5)
**Ethnicity**	
Hispanic or Latino	95 (30.2)
Not Hispanic or Latino	220 (69.8)
**Employment**	
Full time	77 (24.4)
Part time	68 (21.6)
Unemployed	168 (53.3)
**SNAP use**	
Yes	115 (36.5)
No	201 (67.0)
**Food pantry use**	
Yes	35 (11.1)
No	280 (88.9)
**Food security status**	
Food secure	175 (55.6)
Food insecure	140 (44.4)

Abbreviation: SNAP, Supplemental Nutrition Assistance Program.

a Of 414 Fresh Start participants, 315 (76.1%) provided baseline sociodemographic data. All values are number (percentage) unless otherwise indicated.

### Feasibility

Engagement was moderate across programmatic components; of 414 participants, 51.2% (n = 212) attended at least 1 group class, and 39.4% (n = 163) completed at least 1 health coaching assessment and a follow-up encounter, with 2 or 3 group classes or encounters most common. The mean (SD) number of group classes attended was 2.0 (2.9) classes, while the mean (SD) number of health coaching encounters attended was 3.1 (4.5). One-quarter (25.1%; n = 104) met the intervention target of attending 4 or more group classes; 30.2% (n = 125) attended 4 or more health coaching encounters, and few participants attended all 9 group classes (4.3%; n = 18).

### Acceptability

For measures of acceptability, overall satisfaction was 96.5%, with most participants who answered the question on acceptability (n = 146) indicating “very satisfied” (75.3%; n = 110) or “satisfied” (21.2%; n = 31); the remaining 3.4% (n = 5) of participants were neutral. Nearly all group class survey responses indicated that participants learned something new (99.6%; 730 of 733) and increased their confidence and skills (98.5%; 719 of 730). More than half reported purchasing new foods introduced in class (68.0%; 397 of 584), most commonly because of their health benefits (82.8%; 309 of 373) or taste (69.2%; 258 of 373), and most used a recipe at home from a cooking demonstration (72.2%; 419 of 580). Most used all or most of the produce provided (90.6%; 116 of 128) and used program recipes at home (75.0%; 108 of 144). Most participants strongly agreed or agreed that the program improved their blood glucose control (75.3%; 110 of 146), overall diet quality (73.1%; 106 of 145), willingness to try new foods (66.2%; 96 of 145), and access to fresh produce (65.9%; 87 of 132), and that it helped them to become more physically active (56.5%; 82 of 145). Themes and complementary quotes developed from qualitative interviews also support the acceptability of the program among participants (Table 2).

**Table 2:** Open-Ended Themes From Year 2 Interviews (N = 25) of Rural Medically Underinsured Participants With Type 2 Diabetes in the Fresh Start Produce Prescription Intervention, Eastern North Carolina, 2023^a^

Theme	Subtheme	Quote
Mental health and well-being	Health coaching social support	It’s like I had a bond. I have a bond with y’all when y’all do call and talk to us and, you know, y’all text and say, “Hey, how’s your day going?” You know, different things like that. That means a lot to me. Cause I do have my bad days. [AHFC09]
		The life coaching, it really helped me because it made me become accountable. . . . I had goals that I had to meet every week, and I knew that somebody was gonna follow up and made sure, like even when I didn’t feel like doing it, I just pushed myself to get it done. [AHFC87]
		I liked that my health coach had this passion for helping me. . . . Not even my own family members care as much about my health as my health coach did. [SPT05]
	Core team and group classes social support	Well, I wanna say, I want to add their positive attitude. It’s rubbed off on me about getting serious about my health. And I saw it in other people there, too. I could see changes in their attitude. . . . They were trying new foods and becoming engaged about the nutrition. [MERCI24]
		When you get started off with them, you start seeing them around — they become your friends, too. [EPT04]
		Having a group and learning from the group . . . we learned as much from each other as from the class itself. [EPT05]
	Commonality with other patients	I mean, I met people in the class that when I see them in the grocery store, we’ll check each other’s grocery carts sometimes and say, “Is that for you?” . . . And meeting people that are from totally different dynamics than you are from, and they’re, you know, different weights and different heights and different ethnicities and all that. And we all have the same problems. [AHFC05]
		I got a chance to meet some new people, and I just felt like my having diabetes was really a bad thing. I really felt bad about it, but then I met other people, so I’m like, I’m really not the only one, even though I knew that. But it’s just really interesting to see different people, but everybody with the same goal of trying to get them numbers down, get healthier. [AHFC87]
	Ongoing postprogram support	Maybe if we get a reminder email, an encouraging text or email every now and then . . . even kind of send us like a recipe for the month. [WATCH44]
		Even if a health coach calls maybe once or twice a month just to keep encouraging someone. I think that would help. . . . Sometimes people just need encouragement to keep going. A lot of people are living by themselves these days. Just to hear a caring voice encourages people to move or just do something. [HOPE07]
Self-efficacy, clinical, and health behavior improvements	Culinary exposure to new vegetables and cooking methods	The different foods that we tried that I never thought, you know, that I would like, and yeah, so it was really good. [HOPE15]
		I like how they introduced me to more vegetables, some that I probably wouldn’t have eaten before. Before this class I did not know coleslaw was cabbage, but now I like coleslaw more. Usually, coleslaw, I’ll take one bite and then just throw the rest of it away, but I’ve got a new like for vegetables. They’ve introduced me to new vegetables. [WATCH24]
		From the recipes this past spring I’ve developed a good taste for cauliflower and broccoli — stuff I never really ate before. [EPT09]
		I’d like to have more recipes and to learn more about vegetables I haven’t tried before. [SPT03]
	Influencing family and peers	Now my husband is actually eating what I fix and liking it. . . . All my grandkids eat it, they all like it too. [HOPE08]
		I know I’ve made a lot of the recipes, and I’ve even introduced them to my family. [WATCH50]
	Improved food skills and literacy	Yes. So, they showed us how to, you know, count calories and check for different things that have fiber and stuff. And the contents, looking more at the labels, the contents in your food. [WATCH24]
		They taught me how to read the labels. Honestly, I never bothered looking at the labels and, you know, they look, it says how much serving the quantity, this is no good for you. And that I learned through the program, honestly, because I never bothered looking at that before. [WATCH52]
		I learned how to prepare things differently and make them taste better without all the grease and salt. It opened my eyes to what I could actually do at home with what I already have. [AHFC08]
		When I go to the grocery store, I look at everything — the calories, the carbs, the cholesterol — on the items I pick up. [EPT06]
	Observed clinical improvements and lifestyle change	I’ve been avoiding foods high in sugar, carbs, and fat, which has helped a lot with my blood glucose. I eat more vegetables and fruit. [SPT01]
		At one point my diabetes was really bad and I couldn’t see well. Thanks to changes in my lifestyle, I can see better. [SPT02]
		My doctor said everything was great — my A_1c_ was down to 6.1. [EPT02]
		If I keep losing weight, I won’t have to take Metformin anymore. [EPT08]
	Increased motivation and self-efficacy to maintain a healthy lifestyle	It’s really helped. . . . If I don’t take this class, I’ll be sitting around the house, looking at TV, eating a bunch of junk food. I don’t wanna do that. Since I’ve been with the program, I’ve seen a difference in me wanting to be active and want to do more. And I never felt this, y’all helped me be motivated to get out the house, get out my comfort zone, go walk, just going to the mailbox, getting in my car to go to town to go get some groceries or go window shopping. [AHFC09]
		The small group made me comfortable, and the calls kept me accountable. . . . I lost about 30 pounds in a year. [MERCI17]
Preferences and suggestions for program expansion	Desire for continuous resources	Yes. Okay. Thank you. I think that’d be good. A resource place. Or even Facebook. I think Facebook would be good because everybody has Facebook mostly and we can chat with each other. [MERCI17]
		Even if a health coach calls maybe once or twice a month just to keep encouraging someone. I think that would help. . . . Sometimes people just need encouragement to keep going. You know, sometimes with them just sitting around, a lot of people are living by themselves these days. Just to hear a caring voice encourages people to move or just do something. [HOPE07]
	Additional culinary resources	Well, I would like to have more recipes to cook and to learn more about different vegetables that I may have not tried before. This year I learned what collards and kale were. [SPT03]
		I mean if you’ve got diabetes then you’re not supposed to be eating a lot of sweets. So, what can I eat that’s sweet that’s not going to make my blood glucose go through the roof that I can satisfy myself with, you know? [EPT05]
	Additional health education resources	Yes, a stress management class would be very helpful for me right now because I think that is the reason I’ve been having blood glucose problems. [SPT05]
		I also have blood pressure problems, so learning to manage diabetes and high blood pressure would help me. [SPT04]
		It will be helpful to learn more about diabetes medications and how they work. [SPT01]
Overall program satisfaction and synergistic impact	Synergistic and holistic program design	What I like is the four different parts . . . the small groups, the coach, the calls, the produce, all mixed together. It’s rare to find a program like that. I lost about 30 pounds in a year. [MERCI17]
		We would break off into groups and learn together. . . . I’ve never been in anything like that — it was a good experience. [WATCH57]
	Staff cared about patients	To me it was the staff. . . . I didn’t see one staff in there that didn’t act like they cared. I mean, we could be almost whispering among our table, and it was always somebody walking by giving us advice on what we were whispering about. You know, pertaining to the class. So, I really felt good and comfortable. But it’s the staff. I enjoy the food, I enjoy the give outs, but I enjoy the staff the most ʼcause they actually care about what they’re doing. [HOPE07]
		I think, you know, their knowledge base was impressive. And their enthusiasm, you know, their positivity really shined in, they were very encouraging, very kind. [MERCI24]
	Most enjoyed/valued program component	None of us are chef-rated cooks or anything, but they made some really tasty dishes. I like to cook, I like to eat, but I guess my interest in cooking, you know, kind of made that a favorite. [MERCI17]
		It’s hard to choose, ʼcause I’m gonna be honest, it was all good. My stomach wants to say that the foods that I took home and tested. That part I truly love. I mean, ʼcause to be able to, you know, actually try right there some of the recipes that they were sending home with us, you know, that encouraged me more to go home and try those recipes. [WATCH24]
	Group class experience and accessibility	I’m bilingual. I love the fact that they had a translator for people that didn’t speak English. That was absolutely amazing. That doesn’t happen often. So, I really appreciated that a lot, the friendliness, the welcomeness that they made us feel. They were really prepared to answer questions, but they also had the handouts and they were, how can I explain it? They didn’t make it all like fancy speaking, you know, elaborate words. They kept it simple, easy to understand. . . . I love the fact that you even had the papers printed in Spanish also translated. [WATCH52]
		I’m a very hands-on learner and watching something on television is different from being there and being able to do it yourself. . . . Putting your hands on things and even the models that we got to hold and learn about things, that makes things more personal to me. . . . And the ones with you know, the cholesterol and the blood and all that stuff. And the hands-on cooking — that makes a big difference. [AHFC05]

a Information in brackets indicates identification of interview participant.

### Impact

Participants’ food and nutrition knowledge, skills, self-efficacy, and dietary behaviors improved significantly. Diet quality shifted toward increases in healthy food consumption and decreases in processed food consumption. Pooled results across all 3 years showed that of 143 participants, the proportion consuming whole grains 3 to 4 days per week increased from 21.7% (n = 31) to 40.6% (n = 58), while those reporting consuming 5 to 7 days per week increased from 16.1% (n = 23) to 21.0% (n = 30) (*z* = 3.53, *P* < .001). Fruit consumption 3 to 4 days per week increased from 30.8% (n = 44) to 39.2% (n = 56) and 5 to 7 days per week increased from 24.5% (n = 35) to 37.8% (n = 54) (*z* = 4.85, *P* < .001). Vegetable consumption 5 to 7 days per week increased from 26.6% (n = 38) to 51.7% (n = 74) (*z* = 4.93, *P* < .001).

Of 143 participants, those reporting rarely or never consuming processed meats increased from 39.2% (n = 56) to 47.6% (n = 68) (z = −2.74, *P* = .006). Participants reporting rarely or never consuming sweets increased from 41.3% (n = 59) to 62.2% (n = 89) (z = −4.63, *P* < .001). Participants reporting rarely or never consuming sugar-sweetened beverages increased from 55.9% (n = 80) to 69.2% (n = 99) (*z* = −4.32, *P* < .001). Significant reductions were also observed in fried foods: participants reporting rarely or never consuming them increased from 33.6% (n = 48) to 46.9% (n = 67) and those reporting consuming them 3 to 4 days per week decreased from 19.6% (n = 28) to 12.6% (n = 18) (*z* = −2.65, *P* = .008). Regular chips and cracker consumption also significantly decreased, with the proportion of participants reporting rarely or never consuming increasing from 33.6% (n = 48) to 46.9% (n = 67) and consumption 3 to 4 days per week decreasing from 17.5% (n = 25) to 7.0% (n = 10) (*z* = −2.29, *P* = .02). Similar trends were observed when examining yearly stratified outcomes (Table 3A)

**Table 3A:** Preintervention Versus Postintervention Dietary Patterns of Rural, Medically Underinsured Participants With Type 2 Diabetes (n = 143) in the Fresh Start Produce Prescription Intervention, Eastern North Carolina, 2021–2024

Dietary patterns^a^	Response	Year 1			Year 2			Year 3		
		Pre, no. (%)	Post, no. (%)	*Z* (*P* value)^b^	Pre, no. (%)	Post, no. (%)	*Z* (*P* value)^b^	Pre, no. (%)	Post, no. (%)	*Z* (*P* value)^b^
Whole grains	Rarely/never	14 (25.9)	6 (20.0)	.95 (.34)	42 (26.8)	8 (12.3)	3.52 (<.001)	36 (33.0)	11 (22.0)	0.89 (.37)
	1–2 times/week	22 (40.7)	8 (26.7)		56 (35.7)	13 (20.0)		34 (31.2)	10 (20.0)	
	3–4 days/week	12 (22.2)	11 (36.7)		43 (27.4)	28 (43.1)		22 (20.2)	19 (38.0)	
	5–7 days/week	6 (11.1)	5 (16.7)		16 (10.2)	16 (24.6)		17 (15.6)	10 (20.0)	

Fruits	Rarely/never	6 (11.1)	2 (6.5)	2.98 (.003)	31 (19.7)	7 (10.8)	2.21 (.03)	29 (26.6)	3 (6.1)	3.41 (<.001)
	1–2 times/week	16 (29.6)	3 (9.7)		44 (28.0)	12 (18.5)		27 (24.8)	7 (14.3)	
	3–4 days/week	22 (40.7)	10 (32.3)		39 (24.8)	24 (36.9)		24 (22.0)	22 (44.9)	
	5–7 days/week	10 (18.5)	16 (51.6)		43 (25.1)	22 (33.8)		29 (26.6)	17 (34.7)	

Vegetables	Rarely/Never	3 (5.5)	0	2.31 (.02)	25 (15.9)	5 (7.7)	3.63 (<.001)	21 (19.4)	0	2.26 (.02)
	1–2 times/week	15 (27.3)	6 (20.0)		48 (30.6)	6 (9.2)		25 (23.1)	11 (22.0)	
	3–4 days/week	20 (36.4)	5 (16.7)		42 (26.8)	18 (27.7)		33 (30.6)	21 (42.0)	
	5–7 days/week	17 (30.9)	19 (63.3)		42 (26.8)	36 (55.4)		29 (26.9)	18 (36.0)	

Processed meats	Rarely/never	21 (40.4)	11 (35.5)	−0.73 (.47)	69 (43.9)	38 (22.2)	−3.16 (.002)	36 (33.0)	19 (38.0)	−0.05 (.96)
	1–2 times/week	19 (36.5)	17 (54.8)		56 (35.7)	19 (29.2)		45 (41.3)	19 (38.0)	
	3–4 days/week	11 (21.2)	3 (9.7)		24 (15.3)	7 (10.8)		15 (13.8)	8 (16.0)	
	5–7 days/week	1 (1.9)	0		8 (5.1)	1 (1.5)		13 (11.9)	4 (8.0)	

Fried foods	Rarely/never	14 (26.4)	15 (48.4)	−2.04 (.04)	60 (38.2)	27 (42.2)	−1.73 (.08)	38 (35.2)	14 (28.0)	0.04 (.97)
	1–2 times/week	28 (52.8)	12 (38.7)		60 (38.2)	26 (40.6)		42 (38.9)	26 (52.0)	
	3–4 days/week	9 (17.0)	4 (12.9)		25 (14.9)	8 (12.5)		20 (18.5)	6 (12.0)	
	5–7 days/week	2 (3.8)	0		12 (7.6)	3 (4.7)		8 (7.4)	4 (8.0)	

Regular chips and crackers	Rarely/never	21 (41.2)	17 (54.8)	−0.66 (.51)	71 (45.2)	30 (46.9)	−0.91 (.36)	28 (34.9)	19 (38.8)	−1.53 (.13)
	1–2 times/week	23 (45.1)	11 (35.5)		53 (33.8)	26 (40.6)		40 (36.7)	24 (49.0)	
	3–4 days/week	6 (11.8)	1 (3.2)		26 (16.6)	5 (7.8)		19 (17.4)	4 (8.2)	
	5–7 days/week	1 (2.0)	2 (6.5)		7 (4.5)	3 (4.7)		12 (11.0)	2 (4.1)	

Sweets	Rarely/never	19 (35.8)	18 (64.3)	−2.07 (.04)	78 (49.7)	45 (69.2)	−2.78 (.005)	40 (36.7)	25 (50.0)	−2.45 (.01)
	1–2 times/week	20 (37.7)	9 (32.1)		41 (26.1)	17 (26.2)		36 (33.0)	18 (36.0)	
	3–4 days/week	9 (17.0)	1 (1.8)		25 (15.9)	2 (3.1)		18 (16.5)	5 (10.0)	
	5–7 days/week	5 (9.4)	0		13 (8.3)	1 (1.5)		15 (13.8)	2 (4.0)	

Sugar-sweetened beverages	Rarely/never	30 (56.6)	18 (58.1)	0.54 (.59)	79 (50.3)	50 (83.3)	−3.98 (<.001)	65 (59.6)	30 (63.8)	−1.48 (.14)
	1–2 times/week	12 (22.6)	6 (19.4)		35 (22.3)	8 (13.3)		16 (14.7)	13 (27.7)	
	3–4 days/week	8 (15.1)	5 (16.1)		20 (12.7)	2 (3.3)		17 (15.6)	4 (8.5)	
	5–7 days/week	3 (5.7)	2 (6.5)		23 (14.6)	0		11 (10.1)	0	

a Based on Segal-Isaacson et al (42).

b Determined by Wilcoxon signed rank test; *P* < .05 considered significant.

Among the 142 participants with complete food literacy data preintervention and postintervention, we found a mean increase of 7.7 points from 92.6 (SD, 21.6) points preintervention to 100.3 (SD, 21.4) points postintervention (*t* = −4.40, *P* < .001). We observed significant improvements in year 1, from 85.9 (SD, 20.3) points preintervention to 99.6 (SD, 16.8) points postintervention (*t* = −3.71, *P* < .001) and year 2, from 89.1 (SD, 19.5) points preintervention to 100.9 (SD, 19.5) postintervention (*t* = −5.64, *P* < .001) (Table 3B).

**Table 3B:** Preintervention Versus Postintervention Food Literacy of Rural, Medically Underinsured Participants With Type 2 Diabetes (n = 142) in the Fresh Start Produce Prescription Intervention, Eastern North Carolina, 2021–2024

Survey measure	Year 1				Year 2				Year 3			
	No. of respondents	Pre, Mean (SD)	Post, Mean (SD)	*t* (*P* value)^a^	No. of respondents	Pre, Mean (SD)	Post, Mean (SD)	*t* (*P* value)^a^	No. of respondents	Pre, Mean (SD)	Post, Mean (SD)	*t* (*P* value)^a^
Food literacy score^b^	29	85.9 (20.3)	99.6 (16.8)	−3.71 (<.001)	64	89.1 (19.5)	100.9 (19.5)	−5.64 (<.001)	48	99.4 (25.1)	102.2 (21.8)	−0.87 (.39)

a Determined by paired sample *t* test; *P* < .05 considered significant.

b Based on Lavelle et al (43).

### Improvements in HbA_1c_

Improvements in food and nutrition knowledge, skills, self-efficacy, and dietary behaviors translated into significantly improved clinical outcomes through improved glycemic control (ie, HbA_1c_) (Table 4). Across the full analytic sample (n = 225), we found a mean decrease in HbA_1c_ of 0.44%, from 8.06% (SD, 1.96%) preintervention to 7.62% (SD, 1.89%) postintervention (*t* = 4.19, *P* < .001). Consistent with these findings, we found a significant shift toward greater glycemic control in the proportion of participants with controlled HbA_1c_, from 40.4% (n = 91) of participants preintervention to 50.2% (n = 113) postintervention (*P* < .001). Results of multiple linear regression examining the effect of health coaching and group class participation on postintervention HbA_1c_, controlling for baseline HbA_1c_ and sociodemographic characteristics, were significant (*F*_7,178_ = 22.46; *P* < .001; adjusted *R* ^2^ = 0.45). Results for group class participation alone (B = –0.07, *P* = .06) indicated that for every 1 group class attended, HbA_1c_ decreased by an average of 0.07%, although this result was not significant. Among participants who met meaningful-engagement targets and had data on preintervention and postintervention HbA_1c_ (n = 170), we found a mean decrease in HbA_1c_ of 0.53%, from 8.17% (SD, 2.03%) preintervention to 7.63% (SD, 1.90%) (*t* = 4.10, *P* < .001). We also found a significant improvement in the proportion of participants with controlled HbA_1c_, from 37.1% (63 of 170) preintervention to 48.2% (82 of 170) postintervention (*P* < .001). In repeated-measures ANOVA, we found that participants who did not meet either program target (≥4 health coaching encounters or ≥4 group classes attended [n = 47]) had a mean (SD) HbA_1c_ reduction of 0.53% (1.83%), participants who met only the health coaching target (n = 40) had a mean (SD) reduction of 0.15% (1.61%), and participants who met only the group class target (n = 29) had a mean (SD) reduction of 0.47% (1.13%). These results highlight that all program services showed positive trends in clinically significant HbA_1c_ improvements. However, participants who met both the health coaching and group class targets (n = 54) achieved the largest mean (SD) reduction, 0.86% (1.64%), demonstrating a dose–response benefit on HbA_1c_ for participants with higher levels of engagement.

**Table 4:** Change in HbA_1c_ from Preintervention to Postintervention and Key Predictors of Postintervention HbA_1c_ Among Rural, Medically Underinsured Participants With Type 2 Diabetes (n = 225) in the Fresh Start Produce Prescription Intervention, Eastern North Carolina, 2021–2024

Analysis	Full analytic sample (n = 225)^a^	Meaningful program engagement sample (n = 170)^b^
**HbA_1c_**		
Preintervention, mean (SD), %	8.06 (1.96)	8.17 (2.03)
Postintervention, mean (SD), %	7.62 (1.89)	7.63 (1.90)
Mean change (95% CI)	0.44 (0.23–0.64)	0.53 (0.28–0.79)
*t*_df_.(*P* value)^c^	4.19_224_ (<.001)	4.10_169_ (<.001)
Cohen *d* d	0.28	0.31
**Categorical change, no.^e^**		
Improved	30	24
Worsened	8	5
Controlled	83	58
Uncontrolled	104	83
McNemar *P* value^f^	<.001	<.001
**Key predictors of postintervention HbA_1c_ g**		
Baseline HbA_1c_, B (*P* value)	0.63 (<.001)	0.57 (<.001)
Group class, B (*P* value)	–0.07 (.06)^h^	–0.07 (.08)^i^

Abbreviation: HbA_1c_, glycated hemoglobin A_1c_.

a Participants with complete pre–post HbA_1c_ data, regardless of engagement level.

b Participants who met the minimum intended program dose (≥2 health coaching sessions or ≥1 group class).

c Paired-sample *t* tests compared baseline and postintervention HbA_1c_ in each analytic sample. *P* values 2-tailed; significance set at α = .05.

d Calculated by using paired mean difference; significance set at α = .05.

e Control defined as HbA_1c_ <7.0%. Improved, uncontrolled at preintervention, controlled at postintervention; worsened, controlled at preintervention, uncontrolled at postintervention; controlled, controlled at preintervention and postintervention; uncontrolled, uncontrolled at preintervention and postintervention.

f McNemar test assessed changes in the proportion of participants who achieved clinical control (HbA_1c_ <7.0%) from preintervention to postintervention. *P* values 2-tailed; significance set at α = .05.

g Linear regression models examined the effects of program participation (group class and health coaching sessions) on the dependent variable, postintervention HbA_1c_, while controlling for baseline HbA_1c_, age, sex, and race and ethnicity; only significant predictors are shown.

h Full analytic model: n = 186; *R* = 0.68; *R* ^2^ = 0.47; adjusted *R* ^2^ = 0.45; *F*_7,178_ = 22.46, *P* < .001.

i Per-protocol model: n = 142; *R* = 0.66; *R* ^2^ = 0.44; adjusted *R* ^2^ = 0.41; *F*_7,134_ = 14.90, *P* < .001.

### Formative evaluation

Of the 119 individuals enrolled in year 3 of the program, 70 (58.8%) completed the formative evaluation. Interest in remote education strategies was high for synchronous (97.2%), asynchronous (92.7%), and self-guided program notebooks for education and goal setting (97.1%). Participants also expressed strong interest in online cooking (92.9%), physical activity demonstrations (91.4%), and educational videos via social media (89.7%). Expanded clinical and behavioral health services, including physical therapy (83.1%), dental (83.1%), and mental health care (75.4%), were highly desired (Table 5).

**Table 5:** Preferences, Needs, and Interests for an Enhanced Future Iteration of Fresh Start Produce Prescription for Rural, Medically Underinsured Participants (n = 64) With Type 2 Diabetes in the 2024 Fresh Start Produce Prescription Intervention, Eastern North Carolina, 2021–2024

Survey question	No. (%)
**We are considering putting short 1- to 2-min cooking videos online demonstrating new recipes. Would this be something you would view or like to have as an additional resource?**	
Very interested	42 (60.0)
Interested	21 (30.0)
Somewhat interested	2 (2.9)
Not interested	5 (7.1)
Very interested	42 (60.0)
**Would you like to access these videos through social media like Facebook or Instagram, or through a central Farm2Clinic website?**	
Yes	64 (98.5)
No	1 (1.5)
**Which would you prefer? (select all that apply)**	
Facebook	37 (58.7)
Instagram	5 (7.9)
Website	23 (36.5)
Any	4 (6.3)
All 3	10 (15.9)
**We are considering putting short physical activity demonstrations for strength, flexibility, strength and cardio online. Would this be something you would like to view or have as an additional resource?**	
Very interested	39 (55.7)
Interested	22 (31.4)
Somewhat interested	3 (4.3)
Not interested	6 (8.6)
**Would you like to access these videos through social media like Facebook or Instagram, or go to a central Farm2Clinic website?**	
Yes	64 (100)
No	0
**Which would you prefer?**	
Facebook	35 (55.6)
Instagram	4 (6.3)
Website	23 (36.5)
Any	5 (7.9)
All 3	10 (15.9)
**We are considering making a notebook for all participants that would have all of the education handouts, activities, recipes, and physical activity that everyone would receive when they enroll. Does this sound like something you would be interested in?**	
Very interested	49 (70.0)
Interested	17 (24.3)
Somewhat interested	2 (2.9)
Not interested	2 (2.9)
**Which part do you think you would be the most interested in- the education, activities, recipes, or physical activity handouts?**	
Education	3 (4.4)
Activities	1 (1.5)
Recipes	4 (5.9)
Physical activity	3 (4.4)
All of the above	57 (83.8)
**We are considering offering group classes both in person as well as online. Please let us know what you think about these possible options.**	
**Live participation via Facebook Live or Zoom for remote participation**	
Very interested	33 (45.8)
Interested	13 (19.1)
Somewhat interested	9 (13.2)
Not interested	13 (19.1)
**Recorded classes you could watch later or watch if you missed**	
Very interested	42 (61.8)
Interested	17 (25.0)
Somewhat interested	4 (5.9)
Not interested	5 (7.4)
**Short educational videos from program leaders you could access on a website**	
Very interested	34 (50.0)
Interested	23 (33.8)
Somewhat interested	8 (11.8)
Not interested	3 (4.4)
**Short educational videos from program leaders you could access on social media**	
Very interested	30 (44.1)
Interested	23 (33.8)
Somewhat interested	8 (11.8)
Not interested	7 (10.3)

**Figure 3:**
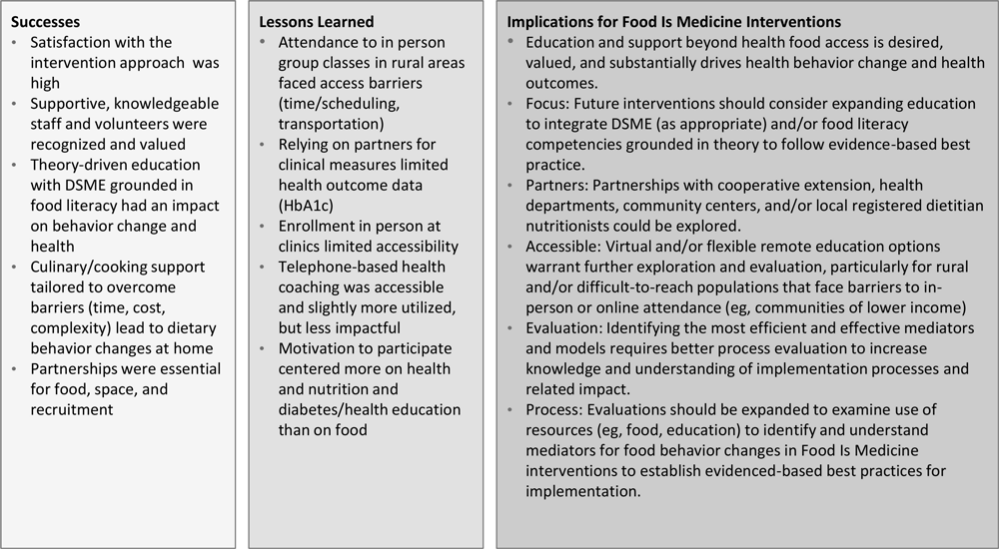
Successes and lessons learned during the Fresh Start produce prescription intervention, or FSPRx, rural eastern North Carolina, 2021–2024, and implications for Food Is Medicine interventions.

We summarized our successes, lessons learned, and overall implications (Figure 3).

**Table:** 

Successes	Lessons learned	Implications for Food Is Medicine interventions
• Satisfaction with the intervention approach was high • Supportive, knowledgeable staff and volunteers were recognized and valued • Theory-driven education with DSME grounded in food literacy had an impact on behavior change and health • Culinary/cooking support tailored to overcome barriers (time, cost, complexity) lead to dietary behavior changes at home • Partnerships were essential for food, space, and recruitment	• Attendance to in person group classes in rural areas faced access barriers (time/scheduling, transportation) • Relying on partners for clinical measures limited health outcome data (HbA_1c_) • Enrollment in person at clinics limited accessibility • Telephone-based health coaching was accessible and slightly more utilized, but less impactful • Motivation to participate centered more on health and nutrition and diabetes/health education than on food	• Education and support beyond health food access is desired, valued, and substantially drives health behavior change and health outcomes. • Focus: Future interventions should consider expanding education to integrate DSME (as appropriate) and/or food literacy competencies grounded in theory to follow evidence-based best practice. • Partners: Partnerships with cooperative extension, health departments, community centers, and/or local registered dietitian nutritionists could be explored. • Accessible: Virtual and/or flexible remote education options warrant further exploration and evaluation, particularly for rural and/or difficult-to-reach populations that face barriers to in-person or online attendance (eg, communities of lower income). • Evaluation: Identifying the most efficient and effective mediators and models requires better process evaluation to increase knowledge and understanding of implementation processes and related impact. • Process: Evaluations should be expanded to examine use of resources (eg, food, education) to identify and understand mediators for food behavior changes in Food Is Medicine interventions to establish evidence-based best practices for implementation.

## Implications for Public Health

The purpose of this study was to evaluate the feasibility, acceptability, and impact of the implementation of the FSPRx intervention among rural, underinsured people with type 2 diabetes. To our knowledge, our evaluation of FSPRx is one of the most comprehensive evaluations of a Food Is Medicine PRx model. This study contributes examples of implementation and evaluation approaches for future Food Is Medicine PRx interventions.

Participants were highly satisfied with the FSPRx intervention. The mixed-methods evaluation showed not only significant improvements in diet quality, food literacy, and health outcomes among participants but also why and how behavior changes were made (eg, enjoying new favorite vegetables, using recipes from a group class cooking demonstration at home, reading food labels while grocery shopping,). Participants who met optimal targets (≥4 group class and ≥4 health coaching encounters) almost doubled mean declines in HbA_1c_ compared with HEALED, which lacked intensive, direct education and behavioral support (0.86% vs 0.47%). Furthermore, among participants who did not meet optimal goals, mean declines of HbA_1c_ were similar (0.44% FSPRx vs 0.47% HEALED), further demonstrating the value of enhanced education and behavioral support.

The context for these behavior changes is important. Participants reported through interviews how they integrated behavior changes into their daily lives, with most describing application of knowledge and skills gained from group class and health coaching support. Group classes, which focused on skill building and increasing health and food literacy and self-efficacy through interactive discussions and activities, physical activity, and cooking demonstrations, were the strongest predictors of clinical improvement. People who attended at least 4 group classes had the greatest improvement; attendance at each group class was associated with a 0.07% decline in HbA_1c_, although this was not significant. Additionally, because group class attendance was the most effective program component, future Food Is Medicine models may consider emphasizing education and collaboration with community partners with expertise in nutrition, food literacy, and culinary knowledge (eg, Cooperative Extension, Expanded Food and Nutrition Education Program [45], Cooking Matters [46]). Research is warranted to identify the most efficient and effective behavioral and educational supports to maximize the impact of Food Is Medicine on health outcomes.

Glycemic control improved during the intervention; however, the feasibility of group education was moderate to low, with only one-quarter of participants meeting the target of attending at least 4 group classes, despite high levels of satisfaction with the intervention. Although the association between the number of group classes attended and postintervention HbA1c did not reach statistical significance in adjusted regression analyses, participants who met both health coaching and group class engagement targets achieved the largest HbA_1c_ reductions, suggesting a potential dose–response benefit of higher overall program engagement. It is important to recognize the unique barriers faced by rural, underresourced communities in attending in-person (eg, transportation) or virtual (eg, internet access) classes layered on top of additional common barriers (eg, scheduling, time). Our formative evaluation allowed us to identify potential areas for improving access to future program. Interest was highest in a self-guided educational notebook and moderately high in virtual options ranging from social media to synchronous or asynchronous online virtual classes. Research is needed to implement and evaluate more accessible educational approaches in rural and underresourced communities.

### Limitations

Our evaluation has several limitations. First was the collection of HbA_1c_ data via retrospective medical record review and reliance on patients’ access and adherence to health care during the intervention. Many participants did not have full preintervention and postintervention HbA_1c_ data, which precluded evaluation of the full sample. Future programs should seek to ensure collection of clinical outcomes during enrollment and postintervention, potentially collecting these data directly instead of relying on patients’ encounters with clinical partners. Second, participants were recruited from and enrolled at clinical partners during face-to-face visits, which resulted in limited attendance and no-shows for approximately half of those scheduled for appointments. Future recruitment and enrollment may benefit from more flexible virtual or telephone-based enrollment. Third, participants were a unique rural, uninsured population with type 2 diabetes, which may limit generalizability of our findings to other populations. Connecting with underinsured or uninsured individuals through a wider network of community partners might strengthen future iterations of this program. Fourth, development and maintenance of undergraduate health science students as health coaches may not be possible for other Food Is Medicine programs; we had access to a large pool of student volunteers. Fifth, the pre–post design cannot determine causality, and while the FSPRx program demonstrated strong effectiveness on participant’s nutrition knowledge, behavior, and health, further research is needed to investigate and establish causality. 

### Conclusion

This research suggests the value of targeted food-centered nutrition and health education, grounded in theory to complement the provision of a PRx to play an essential role in improving diet quality, nutritional status, and health in a rural, underresourced population with type 2 diabetes. The FSPRx model is tailored to overcome barriers that lower-income populations commonly experience (eg, food insecurity, inadequate transportation, internet barriers, lower health literacy, limited space/equipment for cooking and physical activity). Our evaluation approach may be useful for evaluating programs designed for other lower-income and underresourced populations. Satisfaction with our program (acceptability) and impact on nutrition and health were strong; however, attendance and interaction (feasibility) were moderate to limited. Future iterations of the FSPRx program will prioritize developing and investigating approaches to increasing access to educational resources and behavior support to improve engagement and participation. Lastly, a large network of clinical and community partnerships was and will continue to be critical to the implementation of FSPRx. Research is warranted to identify the effective and efficient behavioral and educational supports to optimize impact among program participants as well as to establish robust best practices for the implementation and evaluation of Food Is Medicine programs.
