# Erratum, Volume 23, July 23 Release

**DOI:** 10.5888/pcd23.250471e

**Published:** 2026-09-10

Because of an author error, the name of a coauthor in the article “Evaluation of the 2021–2024 Implementation of the Fresh Start Enhanced Food Is Medicine Intervention Among Rural, Underinsured People With Type 2 Diabetes” was misspelled in the byline. The correct name is Sarah Elliott, not Elliot. The correction was made to our website on August 27, 2026, and appears online at https://www.cdc.gov/pcd/issues/2026/25_0471.htm. We regret any confusion or inconvenience this error may have caused.

